# Ultrabroadband air-dielectric double-chirped mirrors for laser frequency combs

**DOI:** 10.1038/s41377-025-01961-4

**Published:** 2025-08-19

**Authors:** Tianyi Zeng, Yamac Dikmelik, Feng Xie, Kevin Lascola, David Burghoff, Qing Hu

**Affiliations:** 1 Department of Electrical Engineering and Computer Science, Research Laboratory of Electronics, Massachusetts Institute of Technology, Cambridge, 02139 MA USA; 2 General Dynamics Mission Systems, Annapolis Junction, 20701 MD USA; 3 Thorlabs Quantum Electronics (TQE), Jessup, 20794 MD USA; 4 Chandra Department of Electrical and Computer Engineering, Cockrell School of Engineering, The University of Texas at Austin, Austin, 78712, TX USA

**Keywords:** Quantum cascade lasers, Nonlinear optics, Frequency combs

## Abstract

Dispersion engineering is critical for the creation of integrated broadband laser frequency combs. In the long wavelength infrared range (LWIR, 8-13 µm), frequency combs based on quantum cascade lasers are attractive since they are monolithic, fundamental oscillators with high power levels and efficiencies. One effective approach for expanding quantum cascade laser gain bandwidth is by stacking multiple gain media with different center lasing frequencies, as this leads to flatter broadband gain spectra. However, as the gain bandwidth is increased, dispersion becomes the main limiting factor for comb bandwidth. Therefore, achieving broadband combs requires schemes that can flexibly engineer the dispersion over broad bandwidths. Here, we demonstrate the ultimate nanophotonic dispersion compensation scheme: an air-dielectric slab double-chirped mirror, which we fully integrate with the quantum cascade laser gain section. This scheme relies on the highest possible index contrast and therefore provides the maximum correction per unit length over a very broad bandwidth. With this approach, we report the successful demonstration of a broadband room-temperature LWIR laser frequency comb on a gain medium that normally does not form combs without deliberate dispersion compensations. Our air-dielectric mirrors are versatile and can be extended to other integrated laser frequency combs in different material platforms and frequency bands.

## Abstract (web summary)

Using an on-chip dispersion measurement platform, we designed an air–dielectric slab double-chirped mirror integrated into a quantum-cascade laser, achieving room-temperature LWIR combs spanning >100 cm^−1^ at 9.6 µm.

## Graphical abstract

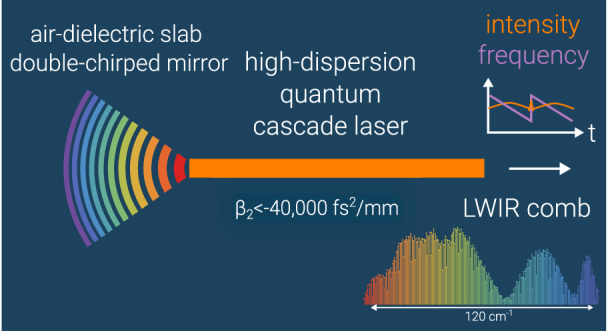

## Introduction

The invention and evolution of optical frequency combs have revolutionized high-precision metrology and spectroscopy^1,2^, among applications including optical communication^3^, ranging^4^, frequency synthesis^5^, optical clocks, and quantum optics. The LWIR range (8–13 *μ*m) is a fertile ground for sensing and spectroscopy because of its richness in spectral fingerprints^1^ and coincides with a transparent atmospheric window. Dual-comb spectroscopy (DCS) using laser combs is perhaps the most actively researched form of direct comb spectroscopy, known for its high frequency resolution, accuracy, and signal-to-noise ratio, all without mechanical motion or bulky dispersive components. Leveraging DCS allows for the development of field-deployable, comb-based sensing and spectroscopy systems for this spectral region. These systems can enable long-range, real-time, multi-species monitoring of target gas molecules^6^. This calls for a compact, broadband, powerful, and ideally mass-producible infrared comb source, and quantum cascade laser (QCL) frequency combs are an ideal solution in this frequency band^7,8^. Unlike other mid-IR comb sources using ${\chi }^{\left(2\right)}$ processes^9,10^, Fabry-Pérot QCL frequency combs rely on an intrinsic ${\chi }^{\left(3\right)}$ nonlinearity originating from the Bloch dynamics^11,12^. Frequency combs based on QCLs were first developed near the LWIR spectral range^7^ using a three-stack gain medium. The gain-induced dispersion (on top of material and waveguide dispersion) severely limits the achievable comb bandwidths.

Several on-chip dispersion compensation schemes have been demonstrated in QCLs, including waveguide engineering^13^, coupled resonators^14,15^, or quasi-phase-matching by attachment of Gires-Tournois Interferometers (GTIs)^16–18^. These approaches are limited in compensation strength either by the tradeoff between waveguide loss and dispersion or by the coupling strength between coupled cavities or waveguides. For GTIs, the intrinsic tradeoff between bandwidth and dispersion magnitude limits the bandwidth over which the dispersion compensation is effective (Supplementary Information A.1). In the terahertz (THz) range, where the material dispersion dominates, a versatile dispersion compensator was successfully developed^19^ based on the principle of double-chirped mirror (DCM) by introducing sinusoidal perturbations to the waveguide width (i.e., using sidewall corrugations). Even though this scheme is based on the same principle of DCM as in Refs. ^20^ and ^21^, the implementation is quite different from the original form. The original DCM was proposed and demonstrated in ultra-short pulse laser systems as a powerful dispersion compensation scheme^20–22^ in the form of thin dielectric layer stacks. The THz corrugated waveguide scheme^19^ is not applicable in the LWIR, as the metals that cover the corrugated sidewalls, which are required for heat removal, cause significant optical losses that prevent the device from reaching the lasing threshold. Furthermore, with a ~10× shorter wavelength, precision electron-beam lithography (EBL) and etching techniques are required to pattern DCM compensators with sub-100-nm lateral resolution and down to ~15-µm depth. Moreover, there is a lack of an easy-to-implement approach for a systematic evaluation of above-threshold QCL laser dispersion in the LWIR, which is essential for effective dispersion compensation, as measurements below the lasing threshold yield quite different information. One could use a THz time-domain spectroscopy setup^23^, but these measurements are more challenging in the mid-infrared at ~10× higher frequencies.

**Fig. 1:**
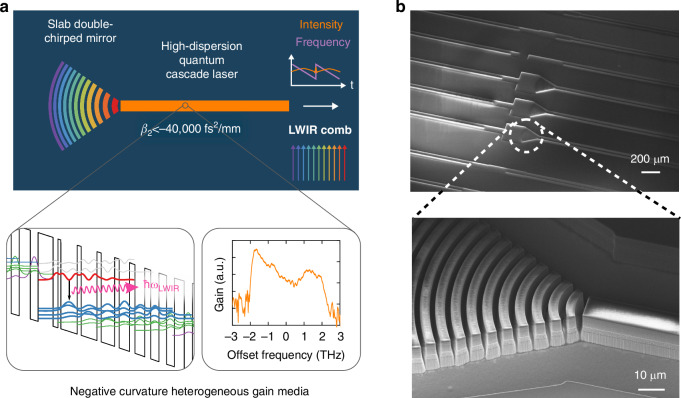
Slab dispersion compensation. **a** A heterogeneous quantum cascade laser (QCL) gain medium has a broadband regime of negative gain curvature and large negative dispersion (β_2_~−40,000 fs^2^/mm), which only manifests itself far above the lasing threshold. By integrating a nanophotonic slab dispersion compensator, which delays short wavelengths relative to long ones, a broadband comb forms. **b** SEM of an actual slab double-chirped mirror, along with an SEM image of curved DCM (prior to gold electroplating for heat removal)

To overcome these challenges, we demonstrate the ultimate double-chirped mirror: air-dielectric stacks (Fig. 1). This structure eliminates metallic losses in the narrow waveguide’s corrugated sidewall, yet it provides the highest dispersion per unit length (stemming from the high index contrast between the semiconductor slabs and air). The DCM structure was fabricated using e-beam lithography and deep dry etching, resulting in the highest aspect ratio of depth/lateral resolution to date. In order to properly account for the complex gain dynamics, we developed an on-chip segmented dispersion measurement platform that is capable of characterizing the above-threshold gain-induced dispersion without a time-domain setup, utilizing the broadband lasing spectrum of QCLs under pulsed bias conditions. With this combined development, we are able to convert an incoherent multimode laser into a broadband frequency comb with a bandwidth of >100 cm^−1^ at a center wavelength of 9.6 µm.

## Results

Dispersion plays a key role in stabilizing and extending the bandwidth of laser frequency combs. Fabry-Pérot (FP) QCL combs, which most naturally operate with a quasi-continuous amplitude^24^ and linearly chirped instantaneous frequency^12,25^, are no exception. Just like their dissipative soliton counterparts in fibers or microresonators^26^, a balance between nonlinearity and cavity dispersion is required to form stable broadband combs. A mean-field theory model^12^ shows that the comb bandwidth of FP QCL combs is inversely proportional to dispersion, on top of other critical parameters such as cavity length, gain curvature, and facet loss. The measurement of above-threshold gain-induced dispersion is a major challenge in the precise dispersion engineering of both interband^27^ and intersubband semiconductor lasers (QCLs)^23^. Historically, semiconductor laser dispersion has been studied with both spectral^28–30^ and time-domain methods^27,31,32^.

Time-domain methods adopt the pump-probe measurement scheme and can be used for both above- and below-threshold measurements of semiconductor laser gain and dispersion. This scheme, however, requires a complicated experimental setup, including the generation of an ultra-short probe pulse, a broadband pump source covering the spectral region of interest, and a nonlinear crystal such as GaSe for detection^33^. Frequency domain methods, on the other hand, require only a spectrometer, a broadband light source, and a linear detector. Below-threshold amplified spontaneous emission (ASE) from the laser cavity can be used as a broadband source to make a laser interrogate itself^28,29^, but is limited in signal-to-noise ratio (SNR) due to the weak emission. More importantly, above threshold, where combs operate and the cavity dispersion can be quite different from that below the threshold, dispersion cannot be obtained this way because an intense lasing signal totally overwhelms the weak ASE signal.

**Fig. 2:**
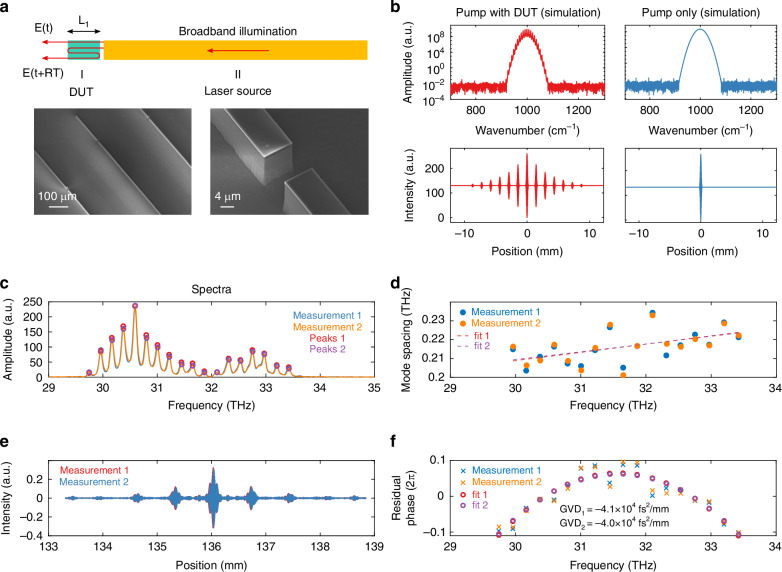
Pump-DUT dispersion characterization platform. **a** Top: Schematic of the segmented dispersion characterization structure. Section II is a pulsed laser source providing broadband illumination, while section I is the DUT. Bottom: SEM images of fabricated DUT-pump devices prior to gold plating. **b** Numerical simulation of interferograms and spectra showing the effect of a dispersive DUT section (for details, see Methods). For simplicity, the pump is assumed to be a Gaussian pulse. The top row shows spectra; the bottom shows interferograms. **c** Spectra from two separate DUT FTIR scans. The DUT has a length of 200 μm and is CW-biased at 12 V, which is far above the 9 V bias at the lasing threshold for a 4-mm-long device. **d** Mode spacing between FP modes, calculated using mode frequencies obtained from **c**. The mode spacing follows a linearly increasing trend with frequency, indicating a negative GVD (Supplementary Information A.6). Higher-order dispersion is present as well, which is the main cause of the fluctuating mode spacing from the linear fit. **e** Interferograms of two consecutive DUT FTIR scans. Trace 2 is slightly shifted to the right for clear visualization. **f** The residual phase exhibits clear negative GVD

In order to accurately determine the laser cavity dispersion under operational bias conditions, we developed a segmented dispersion characterization platform (Fig. 2a) that overcomes all of the challenges discussed above. This scheme leverages integrated photonics technology in the gain material system and combines the pump source and a device-under-test (DUT) on the same chip. In this platform, a QCL biased with short electrical pulses (pulse width 200 ns) serves as the broadband probe light, and alignment is guaranteed by nanofabrication lithography technology. Moreover, the DUT can be biased to a value far above the lasing threshold for a longer cavity (4–5 mm) without lasing. These dispersion characterization devices follow the same continuous-wave (CW) laser fabrication flow for CW comb devices (for details of fabrication, see Methods). This is to ensure that the DUT operates at an identical core temperature to the real devices.

When an IR QCL operates under a pulsed operation regime at room temperature, core temperature fluctuations will lead to a broadband continuous lasing spectrum without resolvable FP laser modes (see Methods and Supplementary Information A.11); this serves as an ideal probing source that is broadband, continuous, and bright. After the probe light enters the DUT, multiple reflections will occur on both facets. These reflected copies of the pump light will manifest themselves as interference echoes on the interferogram collected at the output of a Fourier Transform Infrared (FTIR) spectrometer. Based on a simplified Gaussian pulse, a numerical simulation of the interferogram and spectrum of the pump-DUT device is shown in Fig. 2b (details in Methods), and it qualitatively agrees with the measurement results shown in Fig. 2c and e (details in Methods). In the frequency domain, Fabry-Pérot resonances corresponding to the DUT cavity size and mode index can be observed in the spectrum; their uneven spacing contains dispersion information and can be calculated (Fig. 2c–f, details in Methods) to produce Group Velocity Dispersion (GVD, *β*_2_). In Fig. 2d, the mode spacing increases with frequency, and the fluctuation is mostly due to dispersion at higher orders than GVD.

Repeated measurement confirms an enormous negative GVD value of ~-40,000 fs^2^/mm for this dual-stack gain medium at 12 V CW bias (see Supplementary Information A.8 for dispersion at other biases). This gain medium is designed and optimized for broadband gain coverage without giving any consideration to dispersion. The measured dispersion value is an order of magnitude higher than the combined material and waveguide dispersion and has an opposite sign; it is a direct result of gain-induced dispersion. The sign of the dispersion is consistent with a time-domain spectroscopy measurement result at ~11.7 µm for a single-stack gain medium^33^, with an extracted dispersion of ~-12,700 fs^2^/mm from that measurement. Dispersion measurement data at different DUT biases are shown in Supplementary Information A.8, exhibiting a positive correlation between the magnitude of GVD and bias. Since the DUT is intentionally designed not to lase by itself, its gain is unclamped, and its mode spacing is not influenced by frequency pulling as in a laser cavity. Frequency pulling reduces the unevenness of the mode spacing and makes the cavity appear to be less dispersive. Hence, an effective compensation of the dispersion without frequency pulling (measured here) will result in evenly spaced cavity modes, which makes it natural for a broadband laser to form a comb.

Sidewall corrugations are not applicable as DCMs for IR QCLs because, unlike THz QCLs, their waveguide dimension (~10 µm) is greater than the wavelength in the material (~3 µm). This means the perturbation to the waveguide width has to be strong enough to provide a sufficient index contrast. For a standard waveguide material stack, a transition from a 10 μm width to a 3 μm width (70% change) would only provide an index contrast of ~14%. This would result in elevated sidewall loss in such a narrow region if the sidewall were covered with metals, as in the case of this work for heat removal. Devices with corrugated widths from 3 µm to 10 µm could not achieve lasing at all. On the other hand, if the corrugation expands outward to minimize the sidewall loss, the reflectivity due to the corrugation and introduced dispersion will drop significantly. This inherent tradeoff between optical loss and compensation effectiveness made the corrugated waveguide design impractical for LWIR QCLs.

The operating principle of a DCM is to chirp the frequency of a grating and its amplitude simultaneously in order to reduce the GDD oscillations from impedance mismatch^20^. This is in contrast to singly-chirped dispersion compensators^34–36^. Moreover, double-chirped sidewall corrugations were also implemented for Kerr combs and soliton generation^37,38^. However, none of these works^34–38^ reported the ultimate form of a chirped mirror—an air-semiconductor grating—in which the largest material index contrast can be achieved per unit length. Fortunately, this scheme is workable for LWIR QCLs because the gain medium (from which the electrically isolated DCM is fabricated) has little optical loss at zero bias^39,40^, which is in stark contrast to THz QCLs. The DCM geometry in this work is not flat as in the original work^20^, since the beam is divergent at the long wavelengths of LWIR. Each slab is curved following the phase front of the waveguide mode entering free space. With a high index contrast between Bragg mirror layers, i.e., between the III-V gain medium (${n}_{h}\sim 3.2$) and air (${n}_{l}\sim 1$), this scheme produces a high reflectivity and a substantial amount of GDD. For circular cavity lasers that are sensitive to backscattering, for instance, the quantum walk combs^40^, such a DCM scheme is not applicable.

**Fig. 3:**
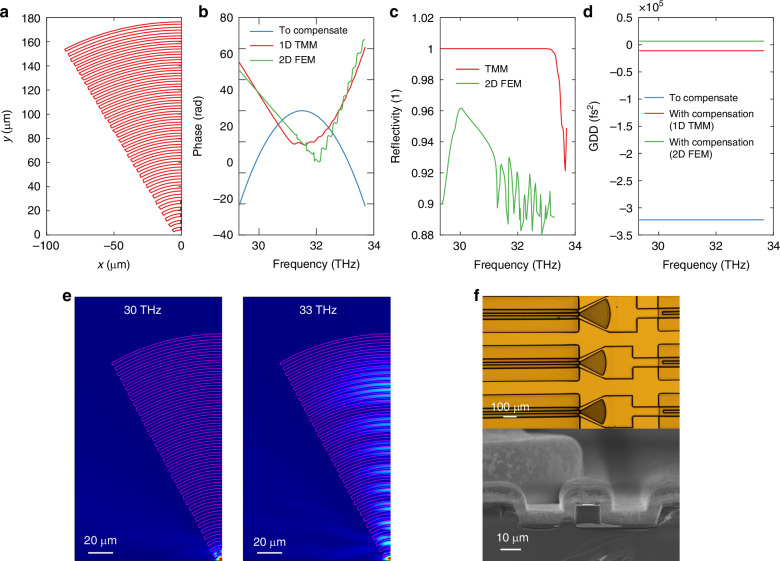
Optimized compensator design in curved geometry. **a** The shape of the curved DCM. **b** Phase plot showing the phase for a 4-mm cavity (blue), representing the phase that needs to be compensated; the round-trip phase of a DCM based on a 1D TMM simulation (red) and a 2D FEM simulation (green). The compensator round-trip phase should have an opposite concavity for proper dispersion compensation. **c** Simulated DCM compensator reflectivity based on 1D TMM (red) and 2D FEM (green). **d** Group delay dispersion of a 4-mm dispersive laser cavity (blue) and a 4-mm dispersive laser cavity with a DCM at the facet (red based on 1D TMM, green based on 2D FEM). **e** Field profile at two frequencies, from low (left) to high (right). Higher frequency modes travel further into the structure, generating positive GDD. **f** Top: Optical microscope image of comb devices with curved compensators after electroplating. Bottom: SEM image of the facet of a mounted, wire-bonded reference laser device that is electroplated with 10-µm-thick gold

We use a total of ten design parameters to uniquely define each DCM design. We implement a 1D Transfer-Matrix-Method (TMM) for coarse search and a 2D Finite-Element-Method (FEM) in the curved Bragg grating geometry (see Fig. 3a) for local optimization of final designs. The dispersion and reflectivity of one final compensator design are shown in Fig. 3b–d. For the negative GVD measured from the DUT characterization (Fig. 2f), the effective cavity length for the higher frequency component should be larger to provide the proper sign of group delay after reflection. Figure 3e captures this trend of frequency-dependent interaction length within the compensator. Figure 3b–d shows a slight deviation of simulation results between 1D TMM and 2D FEM. This is largely because a 1D TMM assumes plane wave propagation and is an idealization of the guided modes in DCM. We use it to efficiently screen millions of DCM designs and then fine-tune the design with the more accurate 2D FEM model. Ultimately 3D FEM should provide the most accurate prediction of device performance, but the required computational resources and simulation time make it impractical as an optimization tool. As an alternative, we conduct 2D FEM in the vertical cross-section to ensure proper mode confinement (Supplementary Information A.9). This result ensures that, in the limit of narrow air gaps, vertical beam divergence becomes negligible and the 3D beam propagation problem can be reduced to a 2D problem using the effective medium approximation. This permits the use of 2D simulations to effectively replace 3D FEM with minimal loss of accuracy. More details on the DCM design are discussed in Methods.

The semiconductor laser frequency comb devices reported in this work are shown in Fig. 1b and Fig. 3f. A major challenge to the fabrication of chirped Bragg reflectors on the QCL platform is the high aspect ratio of vertical etch (15-20 µm) over the lateral dimension with sub-100-nm resolution. Structures with such an extremely high aspect ratio have not been fabricated prior to the present work. We solve this challenging problem through the combination of an optimized EBL process, robust chlorine-based dry etch condition^41^, and a novel hybrid nickel/silicon dioxide etch mask. All room-temperature CW laser devices are patterned, etched, and electroplated with high-reflectivity (HR) coatings on the rear facet and mounted epi-layer-up for room-temperature measurement. Details of the full fabrication procedure can be found in Methods.

**Fig. 4:**
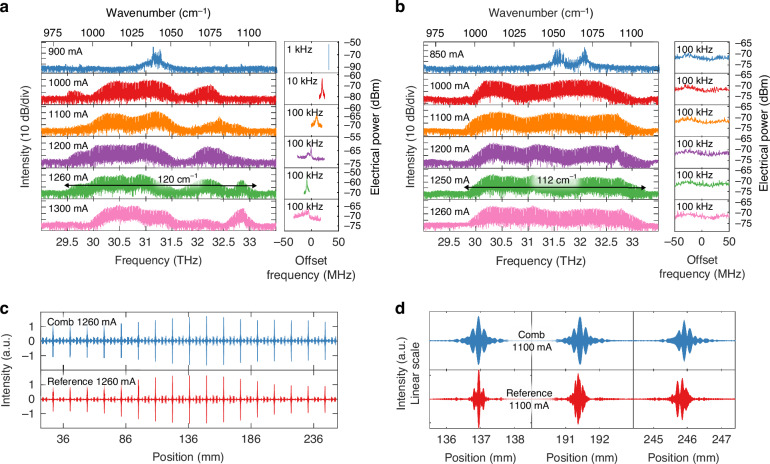
Comb frequency locking results. **a** Spectra and electrical beatnotes of the comb device at various room-temperature CW operating biases. Electrical beatnotes are plotted with a center frequency of 10.99 GHz, the same as for the reference laser. Resolution bandwidths are shown in the top-left corner of the RF spectra. Strong beatnotes can be observed between a bias of 900 mA and 1260 mA as the comb bandwidth continues to increase. Zoomed RF spectra are shown in Supplementary Information A.12. **b** Spectra and lack of electrical beatnotes of a reference laser device at different biases. Even though broadband lasing can be observed above 1000 mA of current injection, up to 112 cm^−1^ at 1260 mA, no sign of a narrow electrical beatnote can be observed at any bias. **c** Interferogram comparison between the comb device and the reference FP laser. The identical interference peak location confirms the matching laser cavity length between the reference laser and the comb device. **d** Interferogram peak comparison from the center burst (left) to the fourth (middle) and eighth (right) echo locations. The echoes of the comb device are identical to the center burst, while the echoes of the reference device deviate significantly from the center burst, implying uneven mode spacings in frequencies

We present clear experimental results of frequency comb generation through tailored dispersion compensation. Lasing spectra and electrical beatnote under room-temperature CW operation are collected and compared between a reference laser without a compensator and a laser comb device with integrated DCM (Fig. 4a, b)). The current-voltage-light characteristics of the device are shown in Supplementary Information A.7. Both devices have an HR coating at the cavity’s rear facet and identical length (4 mm), as affirmed by the matching interferogram peaks in Fig. 4c. These two devices were also placed close to each other on the die to minimize any spatial variation of fabrication conditions. The front facet of the reference device is dry-etched with no extra coating, while for the comb device, DCM Bragg mirrors are fabricated and function as the front reflector. Light is collected at the front facet of both devices. Details of the measurement setup are discussed in Methods. Even though both devices exhibit broadband lasing, no electrical beatnote was observed throughout the entire bias range from the reference device. In contrast, a strong electrical beatnote can be observed from the comb device above the threshold. At a bias of 1260 mA, the comb device has a coherent bandwidth of over 100 cm^−1^ (over 83.7% of the gain bandwidth shown in Fig. S4). The broadest lasing bandwidth of 112 cm^−1^ for the reference device is observed at 1250 mA (but not a comb).

Both devices enter a broadband lasing regime not far above the threshold. At a bias of 900 mA, we observe a narrow comb bandwidth of 6 cm^−1^ with an instrument-limited electrical beatnote full-width-half-maximum (FWHM) of 1.5 kHz. The lasing bandwidth expands with higher injected current and eventually shrinks at an elevated injection level due to thermal rollover (see Fig. 4a 1300 mA vs. 1260 mA bandwidth). Similarly, beatnote bandwidth broadens between 900–1100 mA bias region and then enters a multi-beatnote regime at the bias of 1200 mA. Upon further increase, a 600-kHz FWHM beatnote is observed at 1260 mA with the broadest comb bandwidth. The beatnote eventually disappears close to the thermal rollover point, accompanying the shrinking of lasing bandwidth (1300 mA).

The noticeable contrast between the spectral envelope at comparable current densities confirms the strong coupling and effective feedback from DCM. Both the magnitude and phase of DCM’s reflection will determine the final stable comb state at a given operating condition (gain bandwidth and curvature). The lasing modes between 29.5 and 29.7 THz can only be observed in a compensated comb device; we attribute this to the high reflection band from the compensator (Fig. 3c). Meanwhile, the oscillation in phase at ≥31.7 THz (Fig. 3b) can lead to a spectral envelope dip in this region for the comb device. Such oscillations in reflectivity and phase agree well with the observation of the early DCM work^21^ and can be solved by careful tuning of the chirping parameter for improved impedance matching between the FP laser cavity and compensating Bragg mirror.

To further corroborate our findings, we demonstrate comb coherence through interferogram center bursts and side echoes in Fig. 4c and d. By definition, a comb spectrum is $S\left(\omega \right)=e\left(\omega \right)\times {\sum }_{n}\delta (\omega -n{\omega }_{o})$ where $e\left(\omega \right)$ is the envelope. From the multiplication-convolution duality of the Fourier transform, its interferogram should be $S\left(\tau \right)=E\left(\tau \right)* {\sum }_{n}\delta (\tau -n{\tau }_{o})$. Therefore, a comb should show an identical pattern at all interferogram peak locations. This is true for comb devices, but for the reference laser device, the shape of the interference patterns changes significantly for different echoes. In Fig. 4c, one can observe the obvious decrease of peak amplitude for the side echoes in the reference laser’s interferogram, while the interferogram of the comb device has a much smaller amplitude decrease and is mostly caused by imperfection in alignment through the FTIR. Figure 4d shows zoomed-in identical echoes for the comb device up to the 8th echo (with a maximum mirror traveling distance of ~11 cm in our FTIR) but highly distorted echoes from the center burst for the reference device. This is a telltale sign of uneven mode spacing. A more definitive way to characterize the comb coherence is SWIFTS (Shifted Wave Interferometry Fourier Transform Spectroscopy)^19^, for which a fast detector is required with a bandwidth greater than the beatnote frequency. However, the reflectivity of the DCM was so high (see Fig. 3c) that very little power was out-coupled into our Quantum Well Infrared Photodetector (QWIP). Therefore, we could not detect a meaningful signal level on our QWIP at RF frequencies, which precluded a SWIFT spectroscopy measurement. For the same reason and also due to lack of resonance in the DCM section, we could not directly characterize the dispersion of DCM using the DUT method shown in Fig. 2. To enhance the output power of the laser comb, the HR coating on the rear facet could be removed, allowing emission to be coupled from that facet. The additional mirror loss can be compensated by employing a longer laser cavity or a DCM with higher reflectivity. Watt-level IR QCL comb devices spanning over 80 cm^-1^ have been demonstrated^42,43^. The gain media stacking approach and robust DCM structure demonstrated in this work could further expand the bandwidth of these devices while maintaining a high output power.

## Discussion

We have demonstrated fully integrated longwave infrared frequency combs spanning over 100 cm^−1^ at a center wavelength of 9.6 µm, which rivals the record bandwidths of on-chip comb sources in the LWIR atmospheric window (8–13 *μ*m). The powerful combination of a simplified on-chip dispersion measurement scheme, a physics-informed design and optimization method, a robust, high-resolution fabrication flow, and most importantly, an ultimate scheme of slab DCM compensators without tradeoffs of either gain or bandwidth, makes this framework widely applicable to many other on-chip gain medium platforms to improve the bandwidth of nonlinear photonic devices. For example, the application of the versatile DCM scheme, which has the flexibility to compensate for strong dispersion over a very broad bandwidth, on a gain medium with a much broader gain bandwidth (~440 cm^−1^)^44^ could yield much broader band frequency combs than that demonstrated in this work. With access to Fe:InP regrowth, a buried heterostructure can be used in which the mode in the DCM will be confined both *laterally* and vertically, making the original DCM^20^ directly applicable and the resulting 1D optimization more accurate, enabling much more refined design optimization. The DCM scheme can also be applied to other systems, including but not limited to quantum dot lasers^45^, interband cascade lasers^46^, rare-earth-doped lasers^47,48^, and lithium niobate^49^. In a broader sense, it is applicable to any material capable of providing parametric gain. Further improvement of the current work will entail the enhancement of output power and the careful dispersion characterization of the designed and fabricated DCMs. One potential approach is to form a small cavity (DUT) with two DCMs placed on both ends and use the same on-chip platform demonstrated in this work to measure the cavity resonant frequency and extract dispersion information.

## Materials and Methods

### Design of pump-DUT dimension and measurement procedure

As shown in Fig. 2a in the main text, broadband illumination from section II is coupled on-chip to the short DUT (section I). Since both facets of section I are partially reflective, the probe light will travel many round trips inside the DUT cavity. For simplicity, only one round-trip copy is shown in the illustration. These reflected copies of broadband illumination (pulses) all carry different group delays proportional to the number of round trips traveled inside the DUT cavity. They will then interfere when the moving mirror of FTIR arrives at specific echo locations, causing distorted interference peaks (see Supplementary Information A.4.). Dispersion can be calculated from the collected interferogram.

The measurement is conducted using the following procedure:

- Pulse-bias the pump laser to generate broadband illumination with a continuous spectrum.
- Conduct FTIR measurements and calculate the transmission spectra of light through the DUT (Device Under Test) at a CW bias.
- Process the transmission spectra to calculate GVD. The uneven Fabry-Pérot (FP) mode spacing yields a frequency-dependent mode index, which reflects the dispersion of the biased DUT cavity.

It is imperative that (1) the DUT section is not lasing; (2) the coupling between the DUT and the source laser is not too strong to cause considerable feedback to the pump laser. In terms of device design, these requirements set an upper limit on DUT cavity length and a lower bound on DUT-laser gap size. In practice, a DUT cavity size from 200 to 400 *μ*m is used, and the gap sizes are kept between 10 and 20 *μ*m. Since the DUT has to be biased at the exact operating condition of the actual CW devices, electroplated Au is required for pump-DUT device fabrication, even though the pump laser does not need to be CW-biased.

### Dispersion calculation from DUT FP resonances

Dispersion calculation begins with the measured interferograms. We apply a Hanning window and pad the data 1000 times (add zero arrays 500 times the size of the original data, on both left and right sides) before we apply a Fourier Transform to obtain the transmission spectrum of DUT devices. The scan length of the interferogram for DUT transmission measurement is 8 mm, corresponding to a spectral resolution of 0.625 cm^−1^. This length is chosen so that no observable interference peaks can be observed beyond the scan length. Padding does not increase the spectral resolution of such a transmission measurement but rather improves the peak-finding accuracy. For consistency, we have verified that other peak-finding methods, such as the polynomial fit of transmission peaks, yielded identical results as the zero-padding method. Given this confirmation, finding the FFT of zero-padded interferograms was much faster to perform and, therefore, was used throughout this work.

We then implement a custom peak-finding algorithm to obtain the discrete series of Fabry-Pérot resonances, denoted as $f\left[n\right]$, where n is the mode number. Based on the cavity resonance condition, adjacent FP modes have a round-trip phase difference of 2*π*. We can then create the phase array defined as $\phi \left[n\right]=2\pi n$. Strictly speaking, there is an unknown phase constant ${\phi }_{0}$ that is missing in the expression. However, the linear part of the phase-frequency relation will not affect the dispersion calculation result because it will be removed after the derivatives. For this reason, we set ${\phi }_{0}$ to be 0. The nonlinear $\phi \left[n\right]-f\left[{\rm{n}}\right]$ relation yields the dispersion information. Finally, we arrive at the GVD of the DUT waveguide under operating conditions. In Fig. 2f, we plot the residual phase, which is $\phi \left[n\right]$ with the linear components removed. The curvature of the remaining parabolic curve of $\phi \left[n\right]-f\left[{\rm{n}}\right]$ is proportional to GVD.

For device design, laser cavity Group Delay Dispersion (GDD) is calculated by multiplying the measured GVD by the cavity length. We adopt the physics convention for the calculation of the spectral phase and all other related physical quantities, including group delay (${GDD}\equiv \frac{{\partial }^{2}\phi }{\partial {\omega }^{2}}$) and group velocity dispersion (${GVD}\equiv \frac{1}{{\rm{L}}}\frac{{\partial }^{2}\phi }{\partial {\omega }^{2}}$).

### Numerical simulation of DUT-pump measurement result

In order to quantitatively understand the effect of Dispersion on DUT transmission spectra and the source of noise in dispersion calculation, we set up a numerical model to simulate the interferograms and spectra for given cavity gain and dispersion values. In this model, we use a pulse source with a Gaussian-shaped power spectral density (PSD). The center frequency is 1000 cm^−1^, the standard deviation is 15 cm^−1^.

The frequency range of the simulation is 800 to 1200 cm^−1^, with 0.2 cm^−1^ spacing between each frequency point. The electric field is calculated by the summation of guided waves (mode propagation constant *β*). During propagation, each frequency component will experience the same gain and loss (simplified in this model), but their accumulated phase is frequency-dependent and given by the DUT GVD. Up to 8 facet reflections are considered, or until intra-cavity power is below 0.1% of the input power.

In the simulation, a constant GVD value is used for the DUT section, which corresponds to a parabolic accumulated phase shape. Gain in the DUT section is introduced as an imaginary mode index. The facet power reflectivity is 0.29, calculated from the waveguide mode index of 3.3. The interferogram is calculated using the sum of the E field from two arms of the FTIR (the auto-correlation); one is fixed, and the other is the one with moving mirrors producing the time-delayed/advanced copy of the E field. For the case without DUT, only the coherent lasing E field from the source is considered, along with its time-delayed (advanced) copy from the FTIR moving arm. The simulated interferogram and its FFT without the DUT are shown on the right side of Fig. 2b. When the DUT is introduced to this simulation, multiple copies of the source light will enter the FTIR at different time delays from traveling different numbers of round trips inside the DUT cavity.

In the simplest case, when only one round trip is considered, the added phase of these four fields can be expressed as follows:

$$\begin{array}{ll}{\phi }_{1} & =\,{\phi }_{0}+{\phi }_{{\rm{f}}}\\ {\phi }_{2} & =\,{\phi }_{0}+{\phi }_{{\rm{m}}}\left(x\right)\\ {\phi }_{3} & =\,{\phi }_{0}+{\phi }_{{\rm{f}}}+{\phi }_{{\rm{rt}}}\\ {\phi }_{4} & =\,{\phi }_{0}+{\phi }_{{\rm{m}}}\left(x\right)+{\phi }_{{\rm{rt}}}\end{array}$$

where ${\phi }_{{\rm{f}}}$ denotes the phase accumulated before reflected by the FTIR fixed mirror, ${\phi }_{{\rm{m}}}\left(x\right)$ denotes the phase accumulated before reflection by the moving mirror and is a function of mirror position. ${\phi }_{{\rm{rt}}}$ represents the phase delay from one DUT cavity round trip and is a function of the GVD parameter. All phase parameters are functions of time and frequency.

As expected, with dispersion introduced, the FP mode spacing is not constant. This effect manifests itself in the interferograms as distorted side interference peaks (“echoes”), with increased span in space but decreased peak intensity. This behavior is well captured by this simple yet powerful numerical model; see Supplementary Information A.4. As shown in Fig. 2b without a DUT, we get a single interference peak at the center of the interferogram and a Gaussian spectral profile by taking the Fourier Transform of the interferogram. This is to be expected as the Fourier transform of a Gaussian is also a Gaussian. When DUT is introduced, FP peaks show up as a result of multiple reflections in the DUT. As a result of dispersion, distorted side echoes also show up in the interferogram, as discussed in more detail in A.4.

### Device fabrication

There are several key challenges imposed by the dimension and aspect ratio of chirped Bragg reflectors. First, precise dispersion compensation demands critical dimension variation (CDR) of photonic device features much beyond the resolution of photolithography tools. Electron-beam lithography (EBL) provides sub-100 nm resolution and is implemented to fulfill such requirements. Furthermore, undercut from chemical wet etch^50^ distorts the mask feature and produces a curved sidewall. We adopt chlorine-based dry etch chemistry^41^ and optimize the etch condition for sidewall verticality, smoothness, and minimum micro-loading effect (aspect-ratio-dependent etch rate).

Second, conventional EBL-compatible etch mask (metal) cannot guarantee deep etch (≥15 *μ*m) in narrow unmasked regions (air layer in Bragg reflectors), and the removal of damaged etch mask after the dry etch process is quite difficult. We developed a novel hybrid Nickel/Silicon Dioxide (Ni/SiO2) etch mask (Supplementary Information A.3) for the patterning of laser cavities as well as DCM compensators (also pump-DUT dispersion characterization devices). The fabrication flow chart, including the electroplating process of the thick layer of Au, is illustrated in Figure S1 in Supplementary Information.

The gain medium wafer was epitaxially grown with cladding and active region at Thorlabs; the photonic layer stack consisted of lattice-matched InP/InAlAs/InGaAs. The QC gain medium contains two QC cores with similar designs stacked together. One core has a gain peak roughly at 9.8 μm (30.6 THz), and the other has a gain peak roughly at 9.2 μm (32.6 THz). The ratio of the number of stages of the two cores is 7:5 in order to achieve a gain profile as flat as possible.

Fabrication of the comb device then starts with the deposition of 1.5-2 *μ*m of SiO2 through plasmon-enhanced chemical vapor deposition (PECVD, step (1) in Fig. S1). Negative EBL resist (polymethyl methacrylate, PMMA) is then coated and patterned with optimized dose and proximity effect correction (steps (2) and (3)). 200 nm of Ni hard mask is deposited with an electron-beam evaporation tool and liftoff by stripping the PMMA mask (steps (4) and (5)). We then conduct photolithography on positive photoresist to form the patterns with lower resolution requirements, for instance, the non-device side of the double channel region (see Supplementary Information A.3). The SiO2 layer is then dry etched with CHF3/CF4 chemistry (step (6)). The positive photoresist is removed afterward. This concludes the preparation of a hybrid Ni/SiO2 hard mask for high-aspect-ratio III-V gain material etch.

A dry etch of the cladding and active regions is then conducted in a SAMCO ICP-RIE tool using Cl2/BCl3/SiCl4/Ar gases at 250 °C (step (7)). The pressure, gas ratio, ICP, and RF power are optimized for vertical sidewall and deep etch into narrow air gaps of DCM (with several microns of extra etch into the substrate to reduce mode coupling to the substrate). The damaged hard mask (Ni and SiO2) is then removed with a 30 min immersion in buffered oxide etch (BOE, step (8)). Without the SiO2 underlayer, the damaged Ni mask after the dry etching will be difficult to remove. We use atomic layer deposition (ALD) to grow Al_2_O_3_ as an insulation layer on the sidewall (step (9)), with an estimated optical loss of 2661 cm^−1^ at 10-µm wavelength^51^). Contact windows are opened with thick positive photoresist and 2.5 minutes of BOE etch (step (10)).

Afterwards, a Ti/Au (300/2500 Å) seed layer for electroplating is deposited via electron beam evaporation. 10 *μ*m of electroplated Au is then deposited using Transene TSG-250 solution (step (11)). Plating patterns are defined by thick (>20 *μ*m) positive resist. We then selectively etch Au and Ti to electrically isolate devices on the same chip and to avoid accidental shorting after each device is cleaved (not shown in Fig. S1). Conformal ALD Al2O3 covering the DCM reflectors are also removed with BOE and photoresist mask (see Supplementary Information A.5). The lasing threshold will significantly increase without this step because the Al_2_O_3_ coating the chirped grating mirrors will cause a high optical loss. Notably, since BOE has a substantial undercut, the Al_2_O_3_ covering the first several periods of the grating is chosen not to be removed to avoid device shorting. If we choose to remove Al_2_O_3_ for all grating layers, BOE might affect the Al_2_O_3_ insulation on the sidewall of the main laser cavity, creating an electrical short, thus preventing lasing. We then lap and cleave each die and bond either epi-up or epi-down for pulsed or CW operation. For epi-up mounting, we use indium solder on copper mounts. For epi-down mounting, we use AuSn eutectic solder on AlN submounts.

### Design: double chirped mirror (DCM) Bragg compensator

We describe the generation of Bragg mirror slice thicknesses based on the ten design parameters. We will also explain the choice of parameter range based on physical intuition and coupled mode theory analysis^21^. A total of ten design parameters are involved in creating a double-chirped DCM (as shown in Fig. S2 in Supplementary Information) with enough degree of freedom to act as a robust dispersion compensator. These include:

1. semi-duty-start: ${\eta }_{1}$, starting value of the semiconductor duty cycle.
2. semi-duty-end: ${\eta }_{2}$, ending value of the semiconductor duty cycle.
3. air-T-min: ${T}_{{\rm{air}}1}$, lower bound of the air gap size, mainly due to fabrication constraints.
4. air-T-max: ${T}_{{\rm{air}}2}$, upper bound of the air gap size.
5. dc-m-num: ${m}_{{\rm{c}}}$, total number of grating periods where the duty cycle is chirped.
6. total-m-num: ${m}_{{\rm{tot}}}$, total number of grating periods, has to be larger or equal to dc-m-num.
7. alpha-duty: ${\alpha }_{{\rm{d}}}$, power law coefficient for duty cycle chirping.
8. alpha-T: ${\alpha }_{T}$, power law coefficient for grating Bragg wavelength chirping.
9. lambda-begin: ${\lambda }_{1}$, starting point of grating Bragg wavelength chirping.
10. lambda-end: ${\lambda }_{2}$, finishing point of grating Bragg wavelength chirping.

For each period of the Bragg mirror, the grating period (Bragg wavelength) is determined by:

$$\lambda \left[n\right]=\left[\frac{{\left(\frac{n}{{m}_{{\rm{tot}}}}\right)}^{{\alpha }_{T}}-{\left(\frac{1}{{m}_{{\rm{tot}}}}\right)}^{{\alpha }_{T}}}{1-{\left(\frac{1}{{m}_{{\rm{tot}}}}\right)}^{{\alpha }_{T}}}\right]\left({\lambda }_{2}-{\lambda }_{1}\right)+{\lambda }_{1}$$

where n is the grating (numbering) index, it takes an integer value from 1 to ${m}_{{tot}}$. The semiconductor slab duty cycle is denoted as $\eta \left[n\right]$ and can be calculated using the following relations:

$$\eta \left[n\right]=\left[\frac{{\left(\frac{n}{{m}_{{\rm{c}}}}\right)}^{{\alpha }_{d}}-{\left(\frac{1}{{m}_{{\rm{c}}}}\right)}^{{\alpha }_{d}}}{1-{\left(\frac{1}{{m}_{{\rm{tot}}}}\right)}^{{\alpha }_{d}}}\right]\left({\eta }_{2}-{\eta }_{1}\right)+{\eta }_{1}$$

Here, n only takes integer values from 1 to ${m}_{{\rm{c}}}$. For $n > {m}_{{\rm{c}}}$, $\eta \left[n\right]=0.5$. We enforce this condition in order to form quarter-wavelength Bragg mirrors at the end. Once the Bragg wavelength and duty cycle are determined for each period, the air and semiconductor can be calculated using the following equations:

$$\begin{array}{ll}{T}_{{\rm{air}}}\left[n\right] & =\,\lambda \left[n\right]\left(1-\eta \left[n\right]\right)/2\\ {T}_{{\rm{semi}}}\left[n\right] & =\,\lambda \left[n\right]\eta \left[n\right]/{n}_{h}/2\end{array}$$

where ${n}_{h}$ represents the mode index of the waveguide and is obtained from 2D FEM mode simulation. Notice the extra $1/2$ in the calculation. Finally, we check if the series of ${T}_{{\rm{air}}}\left[n\right]$ lie within the range defined by $\left[{T}_{{\rm{air}}1},{T}_{{\rm{air}}2}\right]$, if not, we normalize ${T}_{{\rm{air}}}\left[n\right]$. Due to fabrication constraints, we set a lower bound for air slab thickness. The etch rate will significantly decrease, and it will also produce a slanted etched facet if the air gap is too narrow. We set the upper bound to minimize mode loss into free space and the substrate.

Given the enormous parameter space, we adopt a hybrid optimization approach. The optimization flow is concisely outlined in Fig. S3 in Supplementary Information. We first use a 1D Transfer-Matrix-Method (TMM) method (details see Supplementary Information A.2) to conduct a coarse search in a larger parameter space and a fine search in a more localized parameter space pinpointed by the coarse search. Two to ten million designs were evaluated in each individual step. The objective function is defined as the difference between group delay dispersion (GDD) provided by the compensator and the required GDD (for a 4 mm laser cavity) to achieve close-to-zero dispersion, as shown in

$${\rm{obj}}\left(\text{design}{\rm{i}}\right)=\mathop{\sum }\limits_{j=2}^{{j}_{\max }}{\left[\frac{\phi \left[j+1\right]-2\phi \left[j\right]+\phi \left[j-1\right]}{\varDelta {\omega }^{2}}-\text{target GDD}\right]}^{2}$$

where ${\rm{i}}$ is the design index, $j$ is the index for frequency sampling points, $\phi \left(j\right)$ is the unwrapped accumulated phase for each frequency point, and $\varDelta \omega$ is the angular frequency spacing between sampling points. The best seed designs serve as starting points for local optimization using 2D Finite-Element-Method (FEM) simulation in the curved Bragg grating geometry (see Fig. 3a). The 2D FEM simulations are approximately two orders of magnitude slower than the 1D TMM. For both 1D TMM and 2D FEM simulation, S-parameter is first calculated. The phase of ${S}_{11}$ is extracted, unwrapped, and differentiated to calculate group delay dispersion, while its amplitude gives the DBR reflectivity (see Fig. 3b–d).

For curved DCM combs, the design parameters are: ${\eta }_{1}=45 \%$, ${\eta }_{2}=55 \%$, ${T}_{{\rm{air}}1}=2\,\mu m$, ${T}_{{\rm{air}}2}=4\,\mu m$, 16 chirped periods, 37 total periods, ${\alpha }_{d}=0.01$, ${\alpha }_{T}=0.01$, ${\lambda }_{1}=36\,\mu m$, ${\lambda }_{2}=30\,\mu m$. For the negative GVD measured from DUT characterization (Fig. 2f), the effective cavity length for the higher frequency component should be larger to provide the proper sign of group delay after DCM reflection. This matches well with this design parameter, where ${\lambda }_{1} > {\lambda }_{2}$, which means a longer wavelength has a shorter effective cavity length. As a matter of fact, all designs in parameter space match this condition based on a physical understanding of this structure. At the end of this DCM design, $\lambda \left[n\right]=30\,\mu m$, and the air slab thickness is 3 times the quarter-wavelength (of 10 *μ*m). This means the DCM design is approximately a doubly-chirped design of a third-order Bragg mirror, which is designed to ease the requirement for lateral resolution in fabrication. DCM curvature follows the phase front of 10-*μ*m-wide waveguide mode emitting into free space. Phase fronts are obtained through a dense 2D FEM simulation.

### Laser comb measurement

All laser devices reported in this paper (combs and reference lasers) are epi-up mounted on a copper mount and placed on top of a TEC (thermoelectric cooler). A thermistor is attached to the copper mount for temperature monitoring and control. Room-temperature CW measurements are conducted at a heat sink temperature of 5 degrees Celsius. The laser signal was coupled into an FTIR system with a ZnSe lens. A liquid nitrogen-cooled mercury cadmium telluride (MCT) detector (IR Associates FTIR-16-0.50) was used to collect light at the output of the FTIR. The detector signals, together with the HeNe interference signals, are both inputted to the computer through a data acquisition system (DAQ). The electrical beatnote is gathered through a bias-T directly from the laser electrical bias channel. The signal is then amplified with two RF amplifiers, providing roughly 40 dB of gain. The RF signal is measured by an HP8592B spectrum analyzer.

### Dispersion measurement

For pump-DUT dispersion measurement, we bias the pump laser using a pulsed voltage driver from Avtech. The driving voltage is selected to produce the broadest and continuous lasing bandwidth. DUT is CW-biased at various voltage levels. The interferogram is collected using an FTIR and MCT in the same way as discussed above for laser comb measurement. A boxcar averager (SR250, Stanford Research Systems) is used for coherent detection of the pulsed signal. For the dispersion measurement result shown in Fig. 2c–e, the pump laser is pulse-biased at 12 V, with a repetition rate of 5 kHz and a pulse width of 200 ns. Its spectrum is shown in Fig. S4 in Supplementary Information, which is continuous over a broad gain bandwidth from 29.5 to 33.8 THz spanning 143.4 cm^−1^. FP modes of the DUT are located with a peak-finding algorithm.

## Supplementary information

Supplementary Information

## Data Availability

The data that support the plots within this paper and other findings of this study are available from the corresponding author upon reasonable request.
