# Supplementary material for: Ultrabroadband air-dielectric double-chirped mirrors for laser frequency combs
Source: Light Sci Appl. 2025 Aug 19;14:280. doi: 10.1038/s41377-025-01961-4 (PMC12365168; doi:10.1038/s41377-025-01961-4)
Supplement: Supplementary file 1 — Supplementary Information [file 41377_2025_1961_MOESM1_ESM.pdf]

Supplementary Information for Ultrabroadband air-dielectric double-chirped mirrors for laser frequency combs

Tianyi Zeng[1], Yamac Dikmelik[2], Feng Xie[3], Kevin Lascola[3], David Burghoff[4], Qing Hu[1*]

[1] Department of Electrical Engineering and Computer Science, Research Laboratory of Electronics, Massachusetts Institute of Technology, Cambridge, 02139, MA, USA.

[2] General Dynamics Mission Systems, Annapolis Junction, 20701, MD, USA.

[3] Thorlabs Quantum Electronics (TQE), Jessup, 20794, MD, USA.

[4] Chandra Department of Electrical and Computer Engineering, Cockrell School of Engineering, The University of Texas at Austin, Austin, 78712, TX, USA.

*Corresponding author: qhu@mit.edu

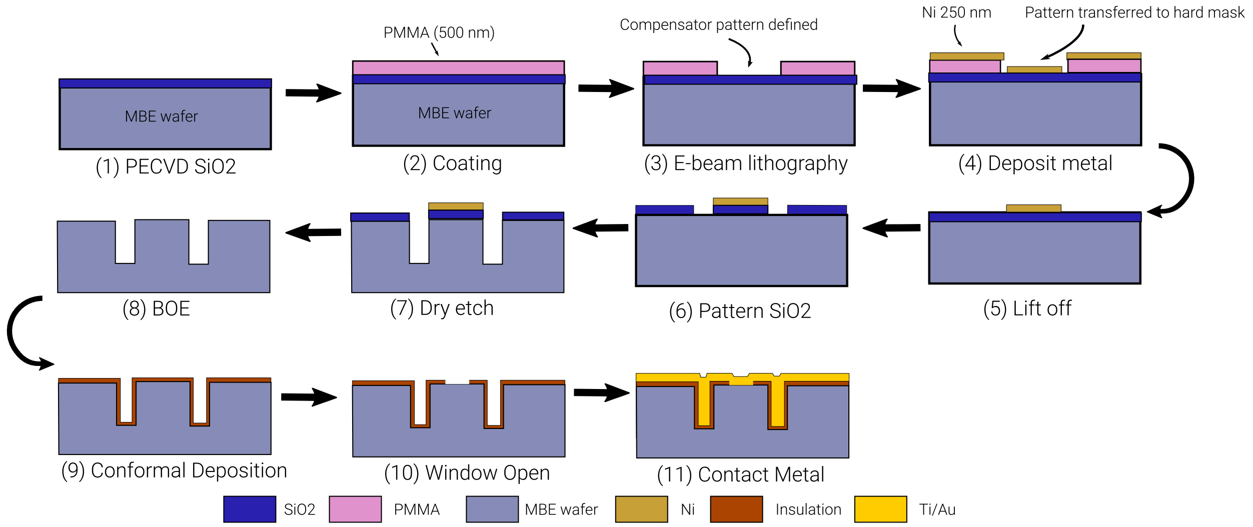

Figure S1. Fabrication flow chart involving both e-beam and photolithography as discussed in Methods: Device fabrication. The view is from the facet of laser ridges.

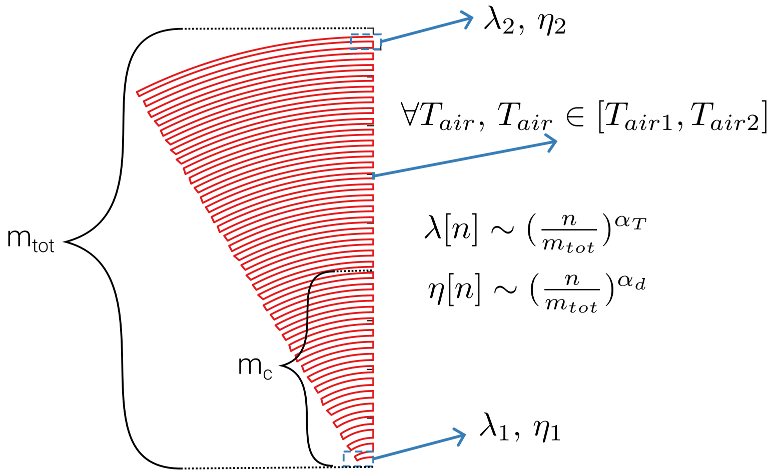

Figure S2. Illustration of the functional role of each DCM design parameter, as discussed in Methods: Design of DCM Bragg compensator.

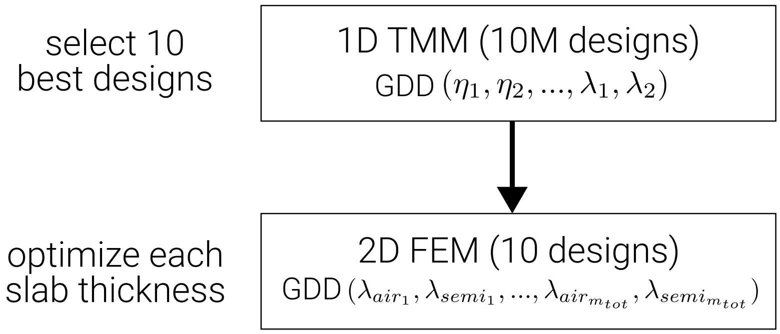

Figure S3. Optimization flow using 1D TMM and 2D FEM as discussed in Methods: Design of DCM Bragg compensator.

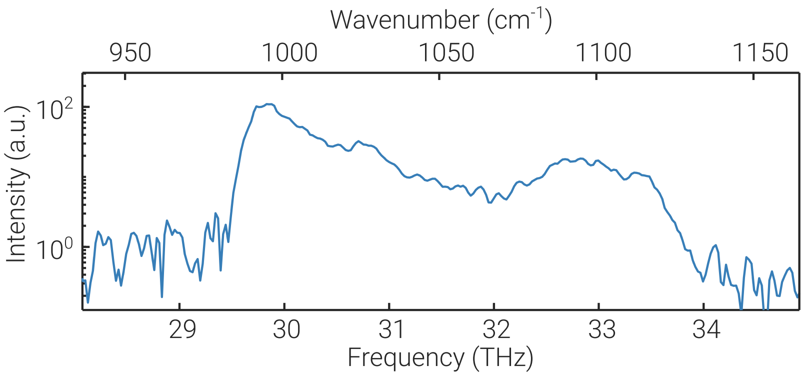

Figure S4. Pulsed spectrum of a 4-mm reference/probe laser biased at 12 V, with a repetition rate of 5 kHz and pulse width of 200 ns, as discussed in Methods: Dispersion measurement.

A.1 GTI simulation

The intrinsic trade-off between flat-band dispersion bandwidth (scales inversely with GTI cavity length) and GDD magnitude (proportional to GTI cavity length) limits its application in broadband laser combs. As numerically shown in Fig. S5, the dispersion bandwidth significantly narrows as the required GDD increases. This feature intrinsically limits the GTI dispersion compensators to narrowband operations.

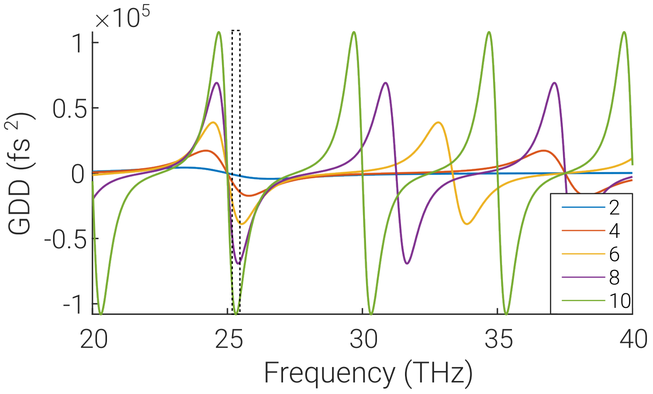

Figure S5. Calculated GDD using 1D TMM for GTI mirror with length ranging from 2 to 10 μm. The facet reflectivity is 25%, and the GTI mirror is composed of an air cavity and a 100% reflectivity mirror. The frequency region with flat GDD suitable for dispersion compensation is highlighted in the dashed box.

A.2 Transfer Matrix Method

For the 1D transfer matrix method (TMM), we simplify the problem to a plane wave traveling in infinite planar slabs of different refractive indices. As mentioned above, all calculations of phase for TMM are **using the physics convention of phase propagation:** $\exp(jkz - j\omega t)$.

The notation for fields at each layer is shown in Figure S6. The wave is propagating from left to right, with FP on the left side as the boundary and air on the right side as the boundary. For the ith layer, A_{i} and B_{i} represents the forward and backward propagating wave at the left side of the layer (the i and i-1 interface). While A_{i}' and B_{i}' represents the forward and backward propagating wave at the right side of the layer and will turn into $A_{\mathrm{i+1}}$ and $B_{\mathrm{i+1}}$ after transmission through the right interface.

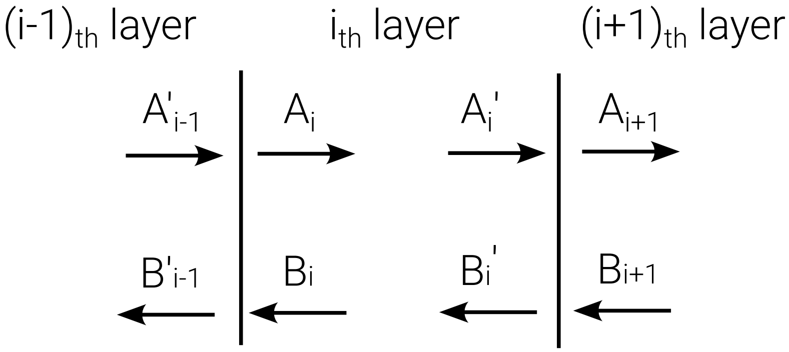

Figure S6. Notation of the field at each interface used in TMM.

At the interface, if the wave is propagating from the ith layer to the jth layer, with the index being n_{i} and n_{j}. We have

$$\begin{bmatrix} A_{\mathrm{i}}' \\ B_{\mathrm{i}}' \end{bmatrix} = \frac{1}{t_{\mathrm{ij}}} \begin{bmatrix} 1 & r_{\mathrm{ij}} \\ r_{\mathrm{ij}} & 1 \end{bmatrix} \begin{bmatrix} A_{\mathrm{j}} \\ B_{\mathrm{j}} \end{bmatrix}$$

where,

$$r_{\mathrm{ij}} = -r_{\mathrm{ji}} = \frac{n_{\mathrm{i}} - n_{\mathrm{j}}}{n_{\mathrm{i}} + n_{\mathrm{j}}}$$

$$t_{\mathrm{ij}} = \frac{2n_{\mathrm{i}}}{n_{\mathrm{i}} + n_{\mathrm{j}}} = 1 + r_{\mathrm{ij}}$$

$$t_{\mathrm{ji}} = \frac{2n_{\mathrm{j}}}{n_{\mathrm{i}} + n_{\mathrm{j}}} = 1 + r_{\mathrm{ji}}$$

The π phase change of the reflected wave propagating from a lower index to a higher index is well captured in this format as a change in sign. Because we are using the physics convention, the field on the left side and the field on the right side of the layer are related by

$$A_{\mathrm{i}}' = A_{\mathrm{i}} exp(jkd_{\mathrm{i}})$$

$$B_{\mathrm{i}}' = B_{\mathrm{i}} exp(-jkd_{\mathrm{i}})$$

In matrix form,

$$\begin{bmatrix} A_{\mathrm{i}} \\ B_{\mathrm{i}} \end{bmatrix} = \begin{bmatrix} \exp(-jkd_{\mathrm{i}}) & 0 \\ 0 & \exp(jkd_{\mathrm{i}}) \end{bmatrix} \begin{bmatrix} A_{\mathrm{i}}' \\ B_{\mathrm{i}}' \end{bmatrix}$$

The sign of phase change from propagation will obviously change if another convention is used, for example, the electrical engineering convention: $\exp(j\omega t - jkz)$. In this model, light comes from the main FP cavity, which has a high index n_h. The light then goes through every period, consisting of air and III-V semiconductor. This corresponds to four matrices for each period: propagation in material 1, interface, propagation in material 2, and the interface. On the other end, the DBR is terminated by an air layer, as is the case for real devices.

The total matrix for n periods should go as

$$M_{total} = I_{hl} \underbrace{P_{1l} I_{lh} P_{1h} I_{hl}}_{\text{1st section}} \dots \underbrace{P_{il} I_{lh} P_{ih} I_{hl}}_{i_{th}\text{ section}} \dots \underbrace{P_{nl} I_{lh} P_{nh} I_{hl}}_{\text{last section}}$$

where I_{hl} means the interface matrix from III-V to air, and I_{lh} vice versa. n sections will need 2n propagation matrices and 2n+1 interface matrices.

The exact relationship of the vectors is:

$$\begin{bmatrix} A_0' \\ B_0' \end{bmatrix} = M \begin{bmatrix} A_n \\ B_n \end{bmatrix} = \begin{bmatrix} a & b \\ c & d \end{bmatrix} \begin{bmatrix} A_n \\ B_n \end{bmatrix}$$

Once a layer sequence for the DBR design is determined, its corresponding total transfer matrix can be calculated as described above, as well as the S-parameters and the accumulated phase over frequencies.

With the phase obtained, GVD can be calculated according to the physics convention as

$$\mathrm{GVD} = \frac{1}{\mathrm{L}}\frac{\partial^2\phi}{\partial\omega^2}$$

A.3 Hybrid Ni/SiO2 Mask

Fig. S7 shows a top view of the device after dry etching (corresponding to step (7) in Fig. S1. The bright part is Ni on top of SiO2 (the blueish part). The area between the two parts consists of trenches deeply etched into the lower cladding InP layer.

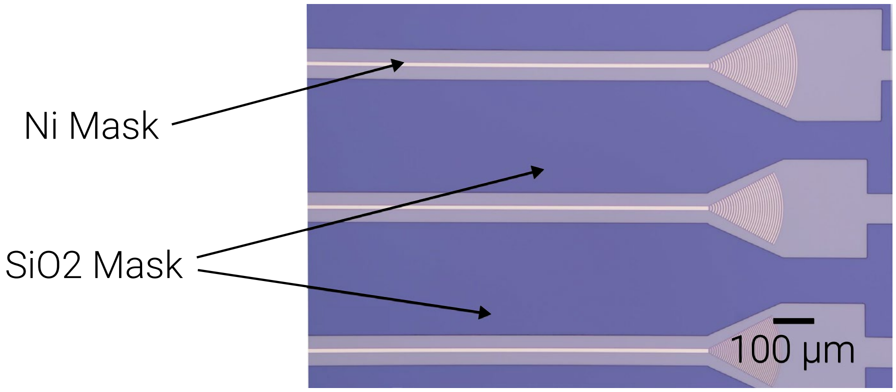

Figure S7. Microscope image showing laser and DCM region defined by Ni/SiO2 hybrid mask, the less critical area formed by a photolithography mask (non-device side of the double-channel).

A.4 Numerical Simulation: Effect of Dispersion

The effect of DUT dispersion on interferograms is shown in Fig. S8 based on the numerical model described in Methods 1.3. The blue trace shows a cavity with no dispersion and with finite gain. Due to facet loss, the cavity has a net loss. That leads to reduced peak intensity at higher-order interferogram peaks for the dispersion-less case, but without lateral distortion. For the red trace, where a large GVD of -50,000 fs^2/mm is introduced, the interferogram peaks not only exhibit decreasing peak intensity but also spread out along the spatial axis. Intuitively, this is a result of different frequency components interfering at slightly different positions.

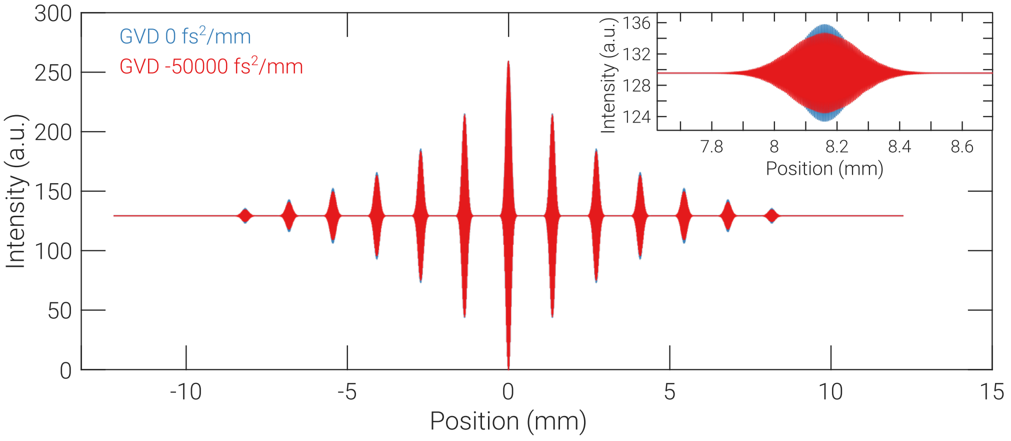

Figure S8. Numerical simulation results of interferograms obtained with different DUT dispersions. The gain is set at 20 cm^{-1}, the DUT length is 400 μm. Inset: Zoom-in at the 6th echo, showing a distorted interference shape (in red) when dispersion is introduced in the DUT cavity.

A.5 Al2O3 removal in DCM region

As mentioned in Methods 1.4 Device fabrication, in the final step, Al_2O_3 was only partially removed to prevent device shorting. Figure S9 shows the comparison between devices with partially removed Al_2O_3 (devices 2 and 4 from left) and devices with full coverage of Al_2O_3 (devices 1 and 3 from left). The BOE solution used for the Al_2O_3 etch has a significant undercut and can cause potential device shorting if the etch window is longer and placed closer to the facet metal pad. We then choose a safe length of unremoved Al_2O_3 to ensure a higher laser yield. For future fabrication optimization, we can develop an isotropic Reactive-Ion-Etch (RIE) recipe that can fully remove Al_2O_3 with much less undercut compared to BOE.

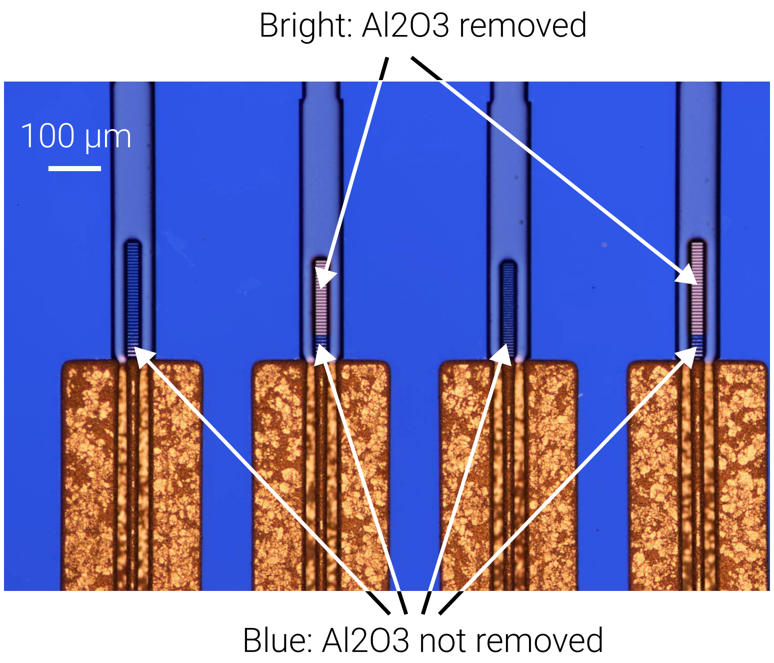

Figure S9. An optical microscope image shows devices with partial Al2O3 removal and no Al2O3 removal. Devices with full Al2O3 coverage on the DCM sidewall show a significantly increased lasing threshold.

A.6 Group velocity dispersion sign in Fig. 2(d)

Group velocity dispersion is proportional to

$$\beta_2 \sim \frac{\partial^2 \phi[m]}{\partial f[m]^2}$$

where m is the mode number. Since the adjacent mode has a fixed phase difference of 2π, GVD is then proportional to

$$\beta_2 \sim \frac{\partial \frac{2\pi}{\Delta f[m]}}{\partial f[m]} = \frac{\Delta \frac{2\pi}{\Delta f[m]}}{\Delta f[m]}$$

Since $\Delta f[m]$ is positive, we then have

$$\beta_2 \sim \Delta \frac{2\pi}{\Delta f[m]}$$

Because $\Delta f[m]$ increases with mode number m, its inverse must be decreasing with m, resulting in a negative derivative. We can reach the conclusion that $\beta_2 < 0$.

A.7 Laser current-voltage-light characteristics

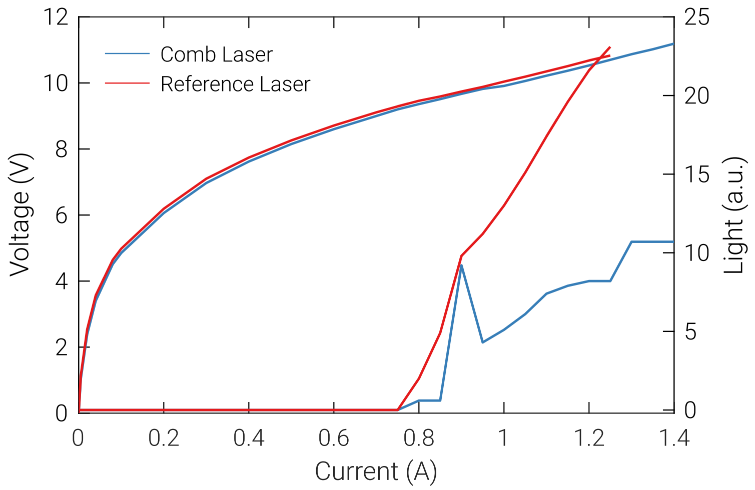

Figure S10. Room-temperature continuous-wave laser operation characteristics for the comb laser and reference laser devices whose spectra are shown in Figure 4.

For the comb laser, a lower lasing threshold is expected due to the reduced mirror loss from DCM. However, for reasons mentioned in Supplementary Information A.5, the unremoved AlO layer close to the facet will induce extra optical loss. Another source of extra loss induced by DCM is mode leakage into the substrate, which we try to minimize with a smaller air gap size. We believe these counteracting effects neutralized one another, resulting in a similar lasing threshold for devices with and without DCMs. For both the comb laser and reference laser, there is a significant

change in laser behavior before and after 900 mA. For the reference laser, the slope efficiency is reduced after 900 mA. This is an intrinsic characteristic of the dual-stack QC gain medium used for this work, accompanied by significant spectral broadening for reference lasers as well as comb devices (Figure 4 and Figure S14).

Furthermore, the comb laser shows elevated lasing power around 900 mA, where narrowband lasing around 31.5 THz with high coherence (beatnote with kHz level FWHM) was observed (Figure 4 in the main text and Figure S14 in Supplementary A.10). After 900 mA, as the comb starts to enter broadband lasing regime, the output power initially drops before gradually increasing again. We believe this unusual light-current curve of the comb laser can be attributed to the strong frequency-dependent feedback of the DCM, its ability to reshape the lasing spectra compared to that of a reference laser, and its interaction with the dual-stack QC gain medium. As a result, the lasing spectra of the comb devices at about 900 mA have an obvious redshift compared to the reference laser, as shown in Fig. 4(a)-(b) in the main text.

A.8 Dispersion measurement at different biases

We show extended DUT dispersion measurement data at different DUT biases (9.6 V and 11.55 V, respectively, in Figures S11 and S12, in addition to the 12 V data shown in Figure 2) to demonstrate strong bias-dependent dispersion of the two-stack QC gain medium. The same DUT and probe laser device are used, with the only difference being the DUT biases. Compared to the dispersion value presented in Figure 2, the gain medium shows a lower absolute value of negative dispersion at lower biases, with a value of about -26,000 fs^2/mm at 9.6 V and -38,000 fs^2/mm at 11.55 V.

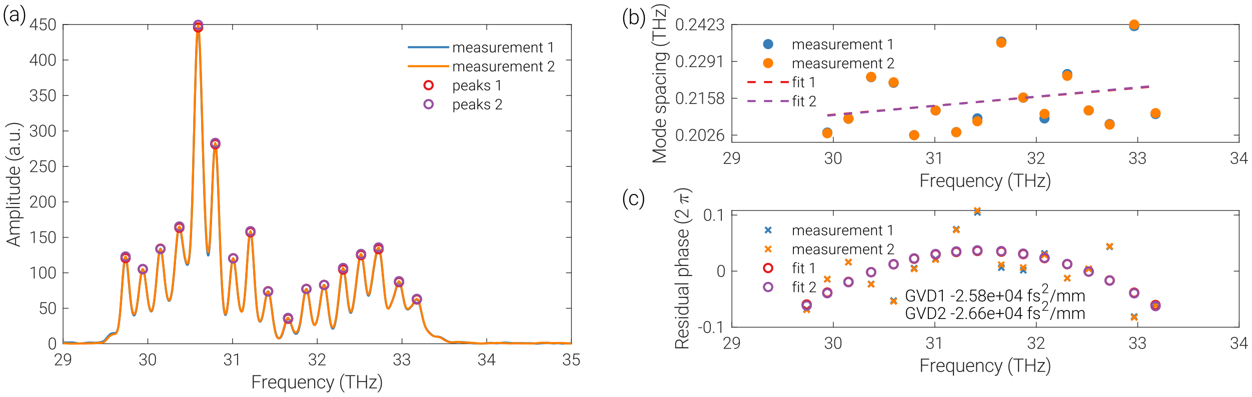

Figure S11. Room-temperature dispersion measurement result of the same DUT device biased at 9.6 V. (a) Spectra from two FTIR scans. (b) Mode spacing between FP modes and linear fit. (c) The residual phase with parabolic fit shows a smaller GVD of around -26,000 fs^2/mm compared to the measured value at 12 V bias condition shown in Fig. 2(f) in the main text.

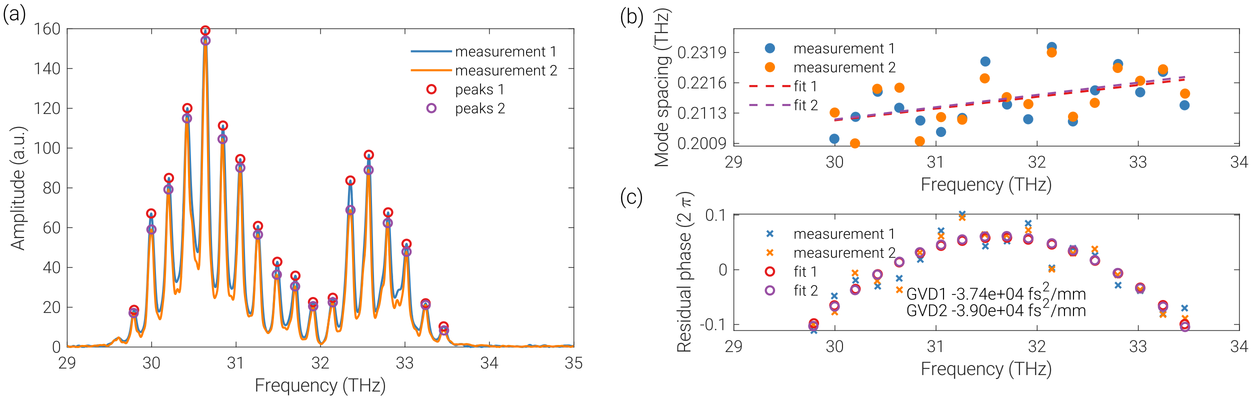

Figure S12. Room-temperature dispersion measurement result of the same DUT device biased at 11.55 V. (a) Spectra from two FTIR scans. (b) Mode spacing between FP modes and linear fit. (c) Residual phase with a parabolic fit, showing a slightly smaller GVD as compared to a 12 V bias condition shown in Fig. 2(f) in the main text, but larger than that of 9.6 V.

A.9 Simulation of the vertical mode confinement in DCM

Strong vertical mode confinement is essential to the effective dispersion compensation of DCM. We adopt two approaches in DCM design and fabrication to avoid mode leakage into the lower cladding: 1) we enforce a narrow air gap size by setting an upper bound of T_{air2}, as mentioned in Methods 1.5; 2) we conduct extra etch beyond the bottom cladding into the InP substrate, as mentioned in Methods 1.4.

Due to limited computational resources, 3D FEM was not conducted on final DCM designs, and instead we conducted 2D FEM simulation in the vertical cross-section (spanned by waveguide longitudinal axis and vertical/growth axis) to verify vertical mode confinement. Figure S13(a) shows a vertical mode profile at three frequencies for the design discussed in Figure 3, with an extra 5 μm etch into the substrate. The proper frequency-dependent interaction length agrees well with the design principle and will generate positive GDD. Figure S13(b) presents a counter-example with larger air gaps and no such extra etch into the substrate. Significant mode leakage into the substrate can be observed.

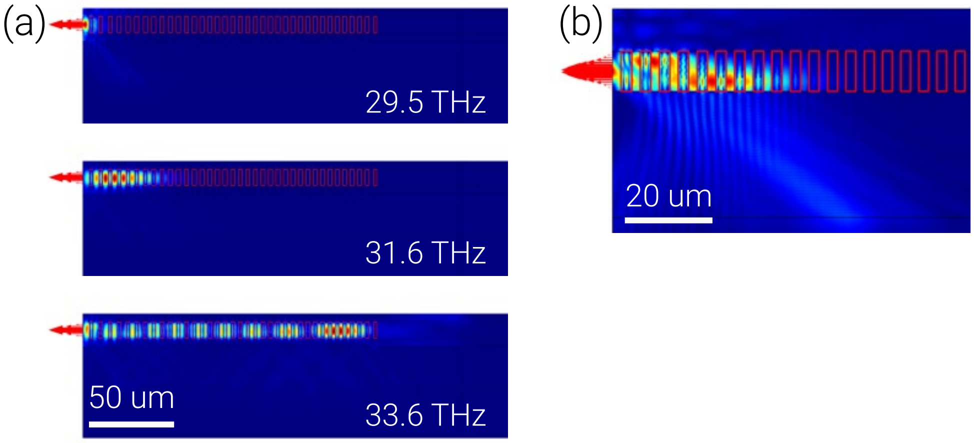

Figure S13. 2D FEM simulation in the vertical domain to show mode confinement. (a) Field profile at three frequencies with the DCM design stacks shown in Fig. 2 and Fig. 4, from low (top) to high (bottom). There is a 5 μm extra etch into the substrate. The

highlighted area is the waveguide core and claddings. Higher frequency modes travel further into the structure, generating positive GDD. Decent confinement is achieved. (b) A field profile at 33.6 THz of another design with large air gaps and no extra etch shows significant mode coupling into the substrate.

A.10 Additional comb device data

The lasing spectra and electrical beatnotes of another comb device with a similar DCM design are shown in Figure S14. The DCM for this device has the same dielectric slab thickness sequence as the one presented in Figures 3 and 4. No curvature was introduced to each semiconductor slab to match the mode phase front. Instead, flat 30-μm-wide slabs are used for each period of the mirror (See Figure S9). At a bias of 1240 mA, a broad comb state spanning 102 cm^{-1} is observed with an electrical beatnote FWHM of 170 kHz. We attribute the narrower comb spectral coverage to the mismatch between the phase front of the emitted light and the DCM reflector shape. Similar to the device shown in Figure 4, at a bias of 900 mA, we observe a comb bandwidth of 26 cm^{-1} with an instrument-limited electrical beatnote FWHM of 2 kHz.

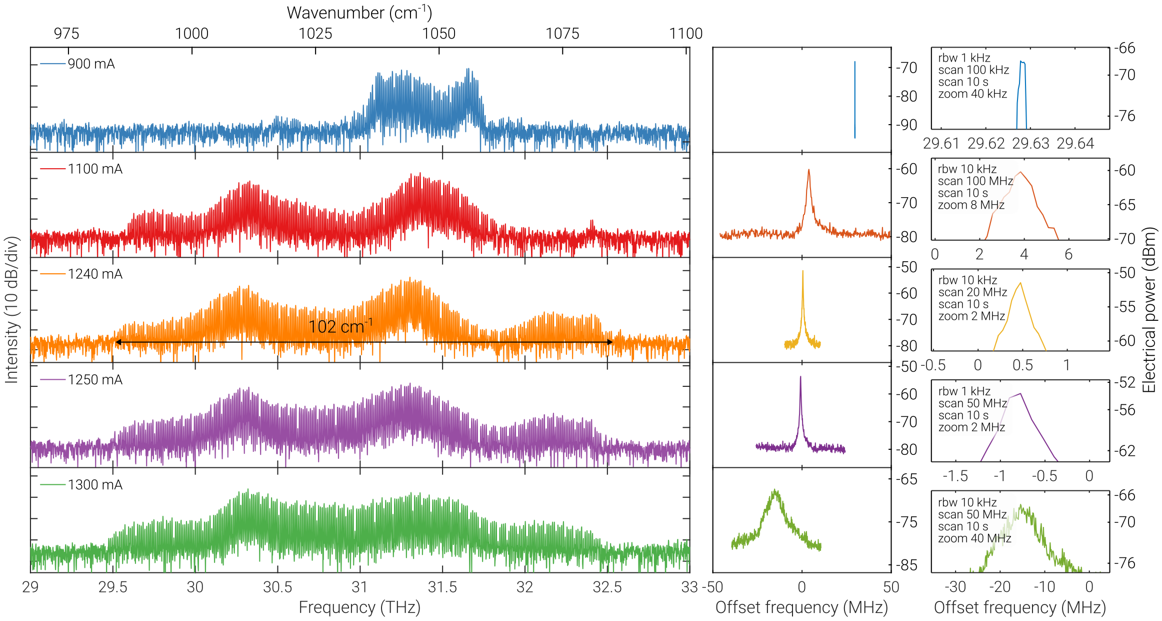

Figure S14. Comb device performance of a similar design to the one shown in Figs. 3 and 4. From left to right, device spectra, beatnote, and zoomed beatnote are shown. The resolution bandwidth, scan span, time, and zoomed span are shown at the top-left corners of the zoomed beatnote plots. At a bias of 1240 mA, the comb device shows a bandwidth of 102 cm-1 and a beatnote full-width-half-maximum bandwidth of 170 kHz.

A.11 Pulsed laser interferograms and spectra at different gate delays

For the DUT dispersion measurement scheme, we use an integrated pulsed QC laser as a bright and broadband source with a continuous spectrum to probe the DUT FP resonances. We show experimental data that when biased with a short electrical pulse (200 ns in the DUT experiment), the pulsed laser source is lasing (still bright) in a fluctuating mode where steady FP modes of its own have not been established. For this experiment, we bias a 4-mm laser cavity of the same gain medium with a 10 kHz repetition rate and a pulse width of 14 μs. We use an MCT (IR Associates FTIR-16-0.50) detector to detect the pulsed signal. A boxcar averager (SR250, Stanford Research Systems) is used to gate the MCT signal with a gate length of 100 ns. The delays are varied by an

increment of 1 μs. The interferograms are shown in Figure S15. At the beginning of the pulse, even at 1 μs, the interferogram shows a single, weaker interference peak at the center, corresponding to a continuous lasing spectrum, as shown in Fig. S4. When the pulse length is longer than 1 μm, the echoes grow noticeably, and the spectrum is no longer continuous.

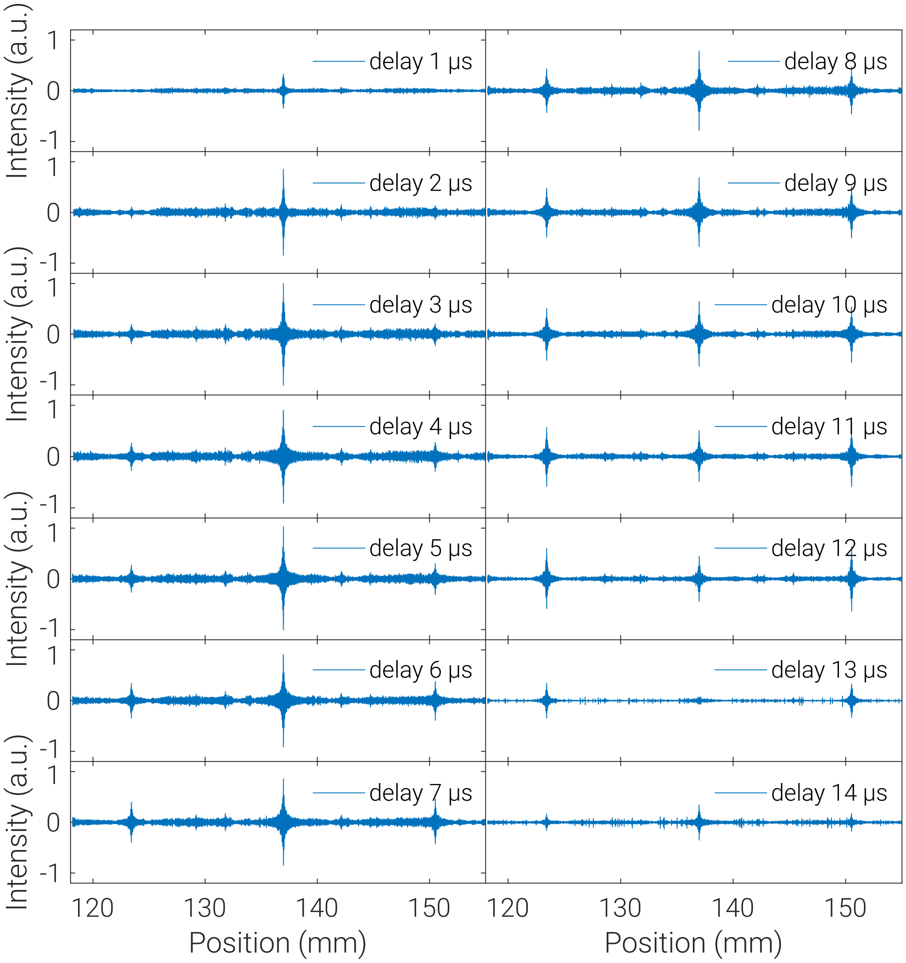

Figure S15. Interferograms with different boxcar gate delays. A 4-mm laser cavity of the same gain medium is biased at a 10 kHz repetition rate and a pulse width of 14 μs, while a boxcar averager is used to gather the data with various gate delays.

A.12 Zoomed beatnote spectra of the comb device from Fig. 4

Figure S16 shows zoomed-in beatnote spectra corresponding to those in Figure 4(a). An instrument-limited electrical beatnote FWHM of 1.5 kHz can be observed at the bias current of 900 mA. The beatnote linewidth then fluctuates as the bias current increases. At the bias current of 1260 mA, where the broadest comb lasing spectra were observed, we chose a zoomed-in span of 2 MHz to show a FWHM of 600 kHz. At a higher injection current, the thermal effect becomes significant, and by 1300 mA, the beatnote nearly vanishes, even though its bandwidth is similar to that at 1260-mA bias.

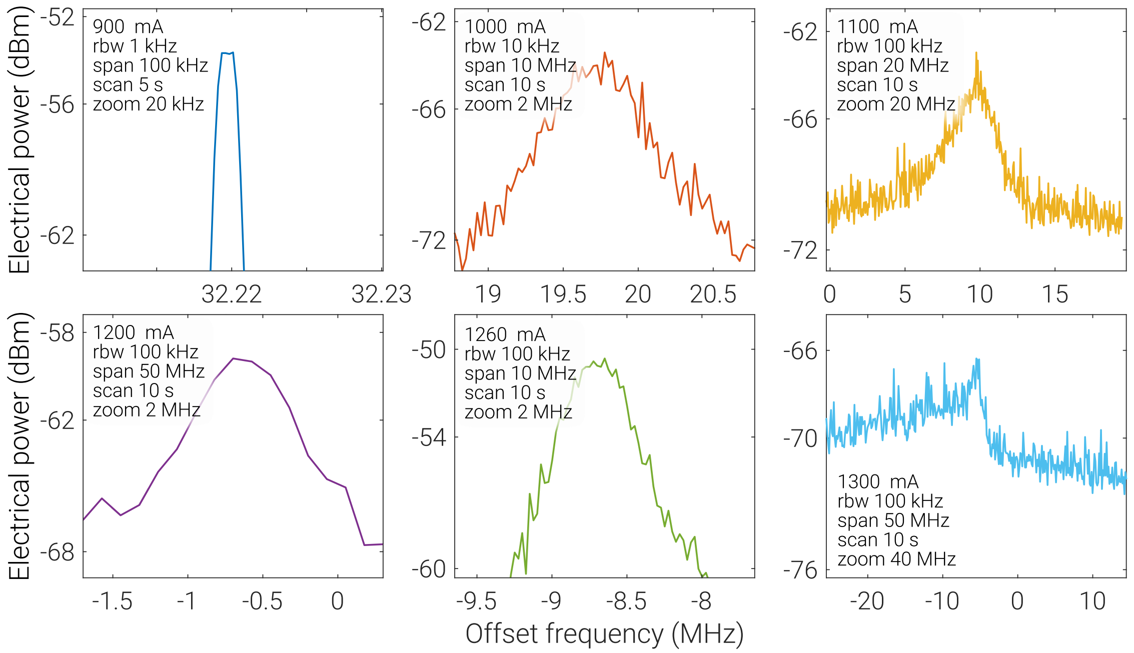

Figure S16. Zoomed-in beatnote spectra of the comb device from Figure 4(a). Bias current, resolution bandwidth, scan span, scan time, and the zoomed-in span of the spectra are shown in plots.
